# Host genetics and susceptibility to congenital and childhood cytomegalovirus infection: a systematic review

**DOI:** 10.3325/cmj.2016.57.321

**Published:** 2016-08

**Authors:** Andrea Gelemanović, Katie Dobberpuhl, Goran Krakar, Inga Patarčić, Ivana Kolčić, Ozren Polašek

**Affiliations:** 1 School of Medicine, University of Split, Split, Croatia; 2 Medical student, Medical College of Wisconsin, Milwaukee, WI, USA; 3 Children’s Hospital Zagreb, Zagreb, Croatia; * These authors equally contributed to the manuscript.

## Abstract

**Aim:**

To summarize available evidence on the role of host genetics in the susceptibility to congenital and childhood cytomegalovirus (CMV) infections by conducting a systematic review of published studies.

**Methods:**

We searched online databases (PubMed, Web of Science, Scopus and HuGe Navigator) for relevant studies with well-defined inclusion and exclusion criteria and assessed the risk of bias using novel Confounding-Selection-Information bias score (CSI).

**Results:**

5105 studies were initially identified, but only 5 met all the inclusion criteria and were analyzed in detail. Polymorphisms of the *toll-like receptors* (*TLR*s) and *mannose-binding lectin* (*MBL*) genes were shown to have an impact on the CMV infection in infants. Polymorphisms of the *TLR2* (rs3804100, rs1898830), *TLR4* (rs4986791), and *TLR9* (rs352140) were shown to have a role in congenital CMV infection. Low *MBL* levels were associated with CMV infection in Chinese individuals, a finding that was not replicated in Caucasians. The overall credibility of evidence was weak.

**Conclusions:**

Based on currently available very limited amount of evidence, it is uncertain whether congenital and childhood CMV infections are under host genetic control. Additional primary studies are needed with more specific research hypotheses that will enable gradual understanding of specific mechanisms of the CMV pathogenesis. More genetic studies in the future will facilitate better understanding of host susceptibility and likely enable novel preventative and curative measures.

Human cytomegalovirus (CMV) is a DNA virus from the *Herpesviridae* family, characterized by a lifelong latency after the primary infection and occasional reactivations in the host. It is one of the most common human infections worldwide, with prevalence varying from 50% of younger to middle-aged women in high-income countries to as much as 90% in low- and middle-income countries (1,2). Such high prevalence is caused by very effective transmission modalities, which include intrauterine, intrapartal, or postnatal courses, as well as exchange through body fluids (3,4). Although most cases are asymptomatic, both in congenital and childhood infections, immunocompromised patients seem to be under the highest risk, where a CMV infection had been reported in as much as 36% of critically ill patients. Additional risk factors included sepsis, transfusions, and the need for mechanical ventilation (5). However, it has been shown that life-threatening complications and life-long sequels in immunocompetent patients are not so rare and that the gastrointestinal and central nervous system were at the highest infection risk (6). Apart from that, CMV has been reported as the most frequent cause of congenital infections, with the prevalence of up to 6.1% (2). The highest risk of fetal infection with CMV occurs during the first half of pregnancy. Primary infections are transmitted more easily and cause congenital damage more frequently than the reactivations (4,7). About 10%-15% of such primary infections turn out to be symptomatic, with jaundice, hepatosplenomegaly, and thrombocytopenia. Over half of these cases will develop neurological impairments, such as sensorineural hearing loss, vision damage, or neurodevelopmental delay. The residual asymptomatic infections, which are more difficult to identify, can also cause these long-term, hampering impairments (1,3,4).

Childhood CMV infections, especially congenital infections, are a very serious public health issue. The socioeconomic impact of a CMV infection is still poorly recognized, even though it is more common than other congenital disorders and it is the major non-genetic cause of hearing loss in children. The development of a CMV vaccine is a high priority as it would substantially reduce the patients’ suffering and the economic cost. It is vital to improve our knowledge and low awareness of congenital CMV since these represent main barriers to wider-scale improvements (8,9). The reason for low awareness is that a CMV infection in immunocompetent adults is usually asymptomatic or mild, thus a primary CMV infection is rarely diagnosed during pregnancy (10). Second, CMV infections may present with delayed onset sequelae (8). Another problem is that the transmission rate for congenital CMV infection is different regarding the maternal infection, where it can be up to 32% in primary and 1.4% in recurrent maternal infections (10). This suggests the hypothesis that maternal immunity provides protection against the congenital CMV infection. Another study has shown that the risk of congenital CMV infection was much higher among seropositive women, as it has been shown that the risk of reactivation or re-infection is much higher in seroprevalent populations (11).

Despite the high burden, there are still no existing effective vaccines. Therefore, preventive measures including prenatal and newborn screening for early CMV virus detection are encouraged. Better understanding of the disease pathogenesis is needed, including prevention of maternal infections and re-infections (8,11).

As it has become evident in the recent years that host genetics plays an important role in susceptibility to various infectious diseases, and considering how widespread CMV infection is and which impact it may have on congenital development, the aim of this study was to perform systematic review of the available evidence of host genetics underlying symptomatic CMV infection susceptibility in the congenital period and childhood.

## Materials and methods

### Literature search

We performed a detailed systematic search to summarize published data related to the role of host genetics in susceptibility to CMV infection. Four sources of information were used: PubMed (*http://www.ncbi.nlm.nih.gov/pubmed/),* Web of Science (*http://wok.mimas.ac.uk*), Scopus (*http://www.scopus.com*), and HuGe Literature Finder (*http://www.hugenavigator.net*). The search was performed on July 20, 2014 (for search terms see Supplementary Table 1)(web extra material 1) and an update was performed on November 2, 2015. Two reviewers independently categorized all identified articles for appropriateness by assessing the title and abstract and the third reviewer made the final selection to resolve any disagreements. Selected articles were read in full by two reviewers independently and evaluated to ensure appropriate qualifications of the inclusion and exclusion criteria. A third reviewer revised all the papers and confirmed adherence to inclusion criteria for the relevant articles. References of the finalized list of relevant articles were checked, and if any appropriate article was missed in the search strategy it was then included. Systematic review was conducted and written under the PRISMA reporting guidelines (12).

### Study selection

To be included in this systematic review, studies had to satisfy several requirements. First, we selected studies that investigated host genetic factors with susceptibility to congenital CMV infections or CMV infections in infants. Second, we were interested in studies based on biallelic single-nucleotide polymorphisms (SNP). We also limited the review to studies published in the English language. Exclusion criteria were animal (non-human) studies, studies based on other genetic marker types (short-tandem repeats or deletions), gene expression studies, adult-based studies, and *in-vitro* studies. Because of our focus on isolated CMV susceptibility, we also excluded studies in which patients were reported to have various concomitant diagnoses and other diseases, studies investigating congenital sensorineural hearing loss, and studies on CMV infection after any type of transplantation.

### Data synthesis and quality assessment tool

Due to the low number of identified articles, we could not perform a quantitative synthesis, but rather three experienced reviewers described the main results and detected problems of each included study. Risk of bias in individual studies was assessed using the novel Confounding-Selection-Information bias score – CSI (13), the elements of which were developed on the basis of several assessment scores, including Venice criteria for assessing cumulative epidemiologic evidence in genetic associations (14), Newcastle-Ottawa case-control scale (15), Cochrane risk of bias tool (16), and previously established quality scores in genetic epidemiology (17). Each of the three domains was graded according to credibility as high (A), intermediate (B), or weak (C). Since information bias domain is divided into three sub-scores, the worst score defined the overall information bias risk. CSI scoring was done independently by two reviewers, while the third reviewer made the final decision in case of any discrepancies. Domains and explanation of CSI grades are listed in Supplementary Table 2.(web extra material 2)

## Results

### Eligible studies

**Figure 1:**
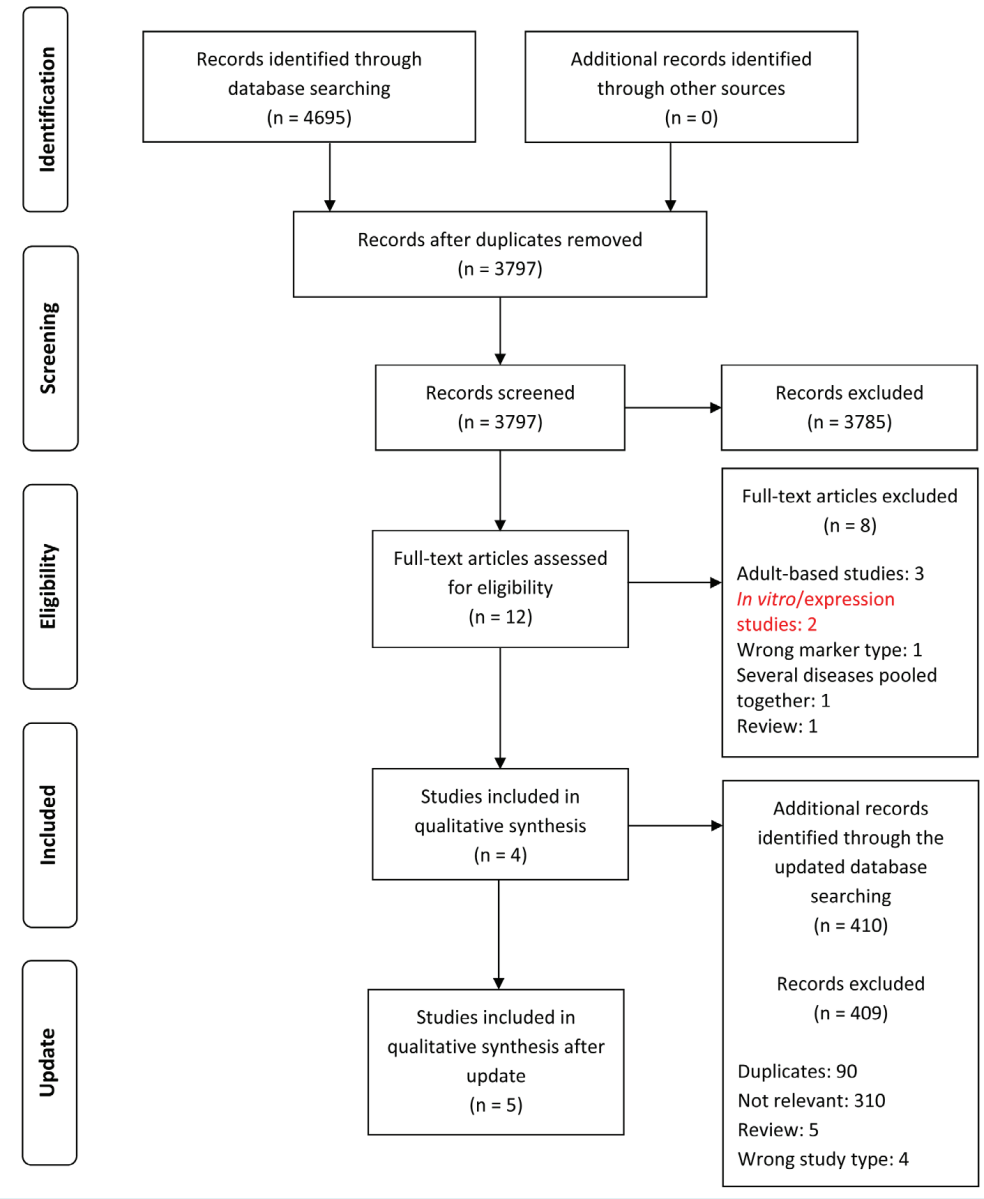
Search strategy flowchart, according to PRISMA guidelines.

4695 articles were acquired in the initial search. After duplicates were removed, the title and abstract of 3797 unique articles were checked for inclusion criteria. The majority of potentially relevant articles were investigating host genetics and susceptibility to CMV infection either after some type of transplantation (n = 26), in patients with another disease (n = 18), or in children with sensorineural hearing loss (n = 7). Among the sub-selected 12 articles (Table 1), only 4 articles met all the inclusion criteria and were considered to be relevant for this study (Figure 1). No further relevant articles were discovered in the reference check of the selected articles. Additional 410 articles were acquired in the updated search, but only 1 met all the inclusion criteria (Figure 1). Key characteristics of the relevant studies are summarized in Table 2. All of the included studies were candidate gene case-control association studies. Selected studies were divided according to the investigated polymorphism with three studies based on *toll-like receptors* (*TLR*s) and two studies on the *mannose-binding lectin* gene (*MBL*). Also, 3 studies investigated genetic susceptibility to CMV infection, while 2 investigated susceptibility to congenital CMV infection. Risk bias of included studies according to CSI scores is presented in Table 3, where all studies have at least one C grade and are considered to have weak credibility. Significant numerical findings from each individual study are summarized in Table 4.

**Table 1:** List of full-text articles assessed for eligibility, with reasons for exclusion

Author, year	Title	Reasons for exclusion
Arav-Boger et al, 2012 (40)	Polymorphisms in toll-like receptor genes influence antibody responses to cytomegalovirus glycoprotein B vaccine	Adult-based studies
Brown et al, 2009 (41)	The R753Q polymorphism abrogates toll-like receptor 2 signaling in response to human cytomegalovirus	*In vitro*/expression studies
Di Bona et al, 2014 (42)	HLA and killer cell immunoglobulin-like receptors influence the natural course of CMV infection	Adult-based studies
Hu et al, 2010 (21)	Association between mannose-binding lectin gene polymorphism and pediatric cytomegalovirus infection	Included
Hurme and Helminen, 1998 (43)	Resistance to human cytomegalovirus infection may be influenced by genetic polymorphisms of the tumor necrosis factor-alpha and interleukin-1 receptor antagonist genes	Adult-based studies
Jablonska et al, 2014 (18)	Relationship between toll-like receptor 2 Arg677Trp and Arg753Gln and toll-like receptor 4 Asp299Gly polymorphisms and cytomegalovirus infection	Included
Muntasell et al, 2013 (44)	NKG2C zygosity influences CD94/NKG2C receptor function and the NK-cell compartment redistribution in response to human cytomegalovirus	*In vitro*/expression studies
Szala et al, 2011 (22)	Mannan-binding lectin-2 (MBL2) gene polymorphisms in prenatal and perinatal cytomegalovirus infections	Included
Taniguchi et al, 2013 (19)	Polymorphisms in TLR-2 are associated with congenital cytomegalovirus (CMV) infection but not with congenital CMV disease	Included
Tao et al, 2012 (27)	Genetic polymorphisms and serum levels of mannose-binding lectin in Chinese pediatric patients with common infectious diseases	Several diseases pooled together
Wujcicka et al, 2014 (23)	Alterations in TLRs as new molecular markers of congenital infections with Human cytomegalovirus?	Review
Zheng et al, 2009 (45)	The HLA-G 14 bp insertion/deletion polymorphism is a putative susceptible factor for active human cytomegalovirus infection in children	Wrong marker type

**Table 2:** Key elements of studies included in the systematic review (CMV – cytomegalovirus; *TLR* – t*oll-like receptors*; *MBL – mannose-binding lectin*; H-W equilibrium – Hardy-Weinberg equilibrium)

Author, year	Gene (rs number)	Cases	Controls	Ethnicity	H-W equilibrium	Adjusted for confounders
Jablonska et al, 2014 (18)	*TLR2* (Arg677Trp) *TLR2* (rs5743708) *TLR4* (rs4986790)	88 infants with CMV infection	28 uninfected infants	Caucasian	not tested	yes (for age and symptomatic infection)
Taniguchi et al, 2013 (19)	*TLR2* (rs3804100) *TLR2* (rs1898830) *TLR4* (rs11536889) *TLR9* (rs352139) *TLR9* (rs352140)	87 infants with congenital CMV infection	83 controls taken from database as general population	Japanese	yes	no
Wujcicka et al, 2015 (20)	*TLR4* (rs4986790) *TLR4* (rs4986791) *TLR9* (rs352140)	18 fetuses and newborns with congenital CMV infection	19 uninfected newborns	Caucasian	yes	no
Hu et al, 2010 (21)	*MBL2* (rs11003125) *MBL2* (rs7096206) *MBL2* (rs7095891) *MBL2* (rs1800450)	104 children with CMV infection	105 uninfected children	Chinese Han	yes	no
Szala et al, 2011 (22)	*MBL2* (different haplotypes)	103 neonates with CMV infection	230 uninfected neonates	Caucasian	not tested	no

**Table 3:** Risk of bias according to CSI score of studies included in the systematic review (CSI – Confounding-Selection-Information bias score; (q)PCR – (quantitative real-time)polymerase chain reaction; QC – quality control)

Author, year	CSI score	Explanation		
		C (confounding bias)	S (selection bias)	I (information bias)
Jablonska et al, 2014 (18)	ACB	A-no apparent confounding	C-no information about controls recruitment	I1:A-cases confirmed by antibody assay and PCR I2:B-antibody negative controls I3:A-genotyping QC reported
Taniguchi et al, 2013 (19)	CBC	C-no information whether controls were matched to cases	B-controls drawn from database (to represent general population)	I1:B-majority of cases confirmed by clinical status and urine-filter based screening, only few confirmed by PCR I2:C-no description of disease status in controls I3:A-genotyping QC reported
Wujcicka et al, 2015 (20)	ABC	A-no apparent confounding	B-hospital based	I1:A-cases confirmed by serological tests and qPCR I2:C-not reported clearly how controls were confirmed as uninfected I3:A-genotyping QC reported
Hu et al, 2010 (21)	ACC	A-no apparent confounding	C-no information about controls recruitment	I1:A-cases confirmed by qPCR and antibody assay I2:B-antibody negative controls I3:C-no genotyping QC reported
Szala et al, 2011 (22)	ABC	A-no apparent confounding	B-hospital based	I1:B-cases confirmed by qPCR and/or antibody assay I2:C-not reported clearly how controls were confirmed as uninfected I3:C-no genotyping QC reported

**Table 4:** Numerical findings from each individual study, only significant results showed (*TLR* – *toll-like receptors*; *MBL* – *mannose-binding lectin*; OR – odds ratio, CI – confidence interval).

					Frequency of significant genotype, %	
Author, year	Gene (rs number), significant genotype	OR [95% CI]	*P*	Model	Cases	Controls
Taniguchi et al, 2013 (19)	*TLR2* (rs3804100), CC	11.7 [1.42–96.30]	0.015	codominant	10.3	1.2
	*TLR2* (rs1898830), AA	0.43 [0.21–0.88]	0.03	codominant	34.5	19.7
Wujcicka et al, 2015 (20)	*TLR4* (rs4986791), CC	1.00 [NA]	0.05	dominant, overdominant	100	77.8
	*TLR9* (rs352140), GA	4.81 [1.14–20.25]	0.05	overdominant	77.8	42.1
Hu et al, 2010 (21)	*MBL2* (rs11003125), H/H	0.28 [0.15-0.55]	0	NA	19.2	45.7
	*MBL2* (rs11003125), H/L	2.51 [1.44-1.40]	0.001	NA	56.8	34.3
	high-*MBL*-level related genotype *YA*-type	0.54 [0.36-0.97]	0.002	NA	47.6	62.9
	low-level related genotype *XB*-type	1.47 [1.10-1.95]	0.007	NA	58.7	40

### Studies referring to SNPs in TLRs

The study by Jabłońska et al (18) investigated SNPs TLR2 Arg677Trp (no reported rs identifier) and Arg753Gln (rs5743708), and TLR4 Asp299Gly (rs4986790) in 88 CMV-infected infants and 28 healthy infants. All CMV-infected infants had the wild-type allele for TLR2 Arg677Trp. Approximately 93% of infected infants did not have TLR2 Arg753Gln SNP and 86% of infants did not have TLR4 Asp299Gly SNP. No significant differences in frequency were observed between TLR2 and TLR4 polymorphisms in infected and healthy infants (18).

In contrast, the study by Taniguchi et al (19) reported that congenital CMV infections were associated with polymorphisms of the *TLR2* gene. The study involved 87 pediatric cases with congenital CMV infection and 83 controls drawn from the database. Cases were grouped into different categories according to the disease severity and the onset of CMV infection. Five SNPs were chosen in the *TLR2* (rs3804100, rs1898830), *TLR4* (rs11536889), and *TLR9* (rs352139, rs352140) gene, which all had more than 5% prevalence in the common Japanese population. Results showed that the CC genotype of TLR2 rs3804100 and the AA genotype of TLR2 rs1898830 were significantly more frequent in infants with congenital CMV infection than in the general population. No significant difference was observed in the frequency of TLR4 and TLR9, and no significant association was found between different groups of infants with congenital CMV disease (19).

Another case-control study of congenital CMV infections and susceptibility to *TLR4* and *TLR9* by Wujcicka et al (20) sampled 18 infected fetuses and newborns and 19 control cases. Half of the infected cases showed symptomatic disease. Two candidate genes were screened for association with congenital CMV infection – *TLR4* (rs4986790, rs4986791) and *TLR9* (rs352140). The main results of this study were that CC genotype at TLR4 rs4986791 and GA genotype at TLR9 rs352140 occurred more frequently among the infected fetuses and newborns, while no significant difference was observed for TLR4 rs4986790. Taking into account the influence of multiple TLR4 and TLR9 SNPs on the development of congenital CMV infection, this study showed that GCA haplotype at rs4986790, rs4986791, and rs352140, respectively, was significantly associated with CMV congenital infection (odds ratio [OR] 6.5 × 10^12^, *P* ≤ 0.0001), and that ACA haplotype was more frequent among symptomatic CMV-infected cases than among asymptomatic cases (*P* ≤ 0.0001) (20).

### Studies referring to SNPs in MBL

Hu et al (21) genotyped 104 CMV-infected children and 105 healthy children for 4 polymorphisms in the *MBL2* gene (rs11003125 -550H/L, rs7096206 -221Y/X, rs7095891 + 4P/Q, rs1800450 + 230A/B). No significant difference was found at the -221 and +230 site. For the -550 region of the promoter, the *H/L* genotype was more frequent in CMV patients, while the *H/H* genotype was more frequent in healthy controls. The frequency of the high-*MBL*-level related genotype *YA*-type was much lower in patients, but the low-level related genotype *XB*-type was more frequent (21).

Szala et al (22) conducted a study to define the correlation between the MBL2 polymorphisms with perinatal and prenatal CMV infections. They included 103 CMV-infected and 230 uninfected neonates, 59 out of which had mothers with CMV infection and 171 had CMV-uninfected mothers. Results showed no significant difference in the polymorphic allele frequencies (A/O + O/O) or with genotypes associated with lower levels of *MBL* (LXPA + O/O), which usually results in the lower MBL protein levels in two control groups. The study also determined there were no significant differences of allele frequencies for the carriers of MBL polymorphisms and for those lacking the MBL protein between the CMV-infected group and controls (22).

## Discussion

This study represents the first systematic field synopsis of all published studies on host genetic factors and susceptibility to congenital and childhood CMV infections. Currently, there is no reliable evidence to either confirm or exclude the association between the tested candidate gene SNPs and CMV infection, due to lack of replication of initial findings and overall weak credibility of available evidence. This conclusion is based on a very limited number of studies, which prevented more advanced analysis and meta-analysis. Each of the identified studies had a specific set of limitations, further contributing to heterogeneity and inability to perform a synthesizing data analysis.

The available studies are based on the genes that were expected to have a role in the disease pathogenesis. *TLR*s are involved in the recognition of the surface glycoproteins of pathogens and activation of the immune system response. Numerous *in vitro* studies confirmed the role of *TLR*s in immunity to CMV infection and it was proven that *TLR4* and *TLR2* recognize CMV. TLR2 Arg753Gln (rs5743708) and TLR4 Asp299Gly (rs4986790) are correlated with a higher risk of acquiring CMV infection and disease in transplant patients (23-26). *TLR2* polymorphisms might have been involved even in CMV transmission from infected mothers to the fetus (19). But, findings from infant studies on this polymorphism are contradictory. A study on Caucasian infants showed no association since similar *TLR* genotype frequencies were observed in CMV-infected and healthy infants (18). Interestingly, an association was observed in adults, where wild-type TLR2 Arg677Trp SNP showed an increased risk and heterozygote TLR2 Arg677Trp (no reported rs identifier) a decreased risk of CMV infection (18). On the other hand, SNPs rs3804100 and rs1898830 in *TLR2* were observed at a higher frequency in Japanese infants with congenital CMV infection (19). Reasons for these differences might come either from ethnicity (Caucasian vs Japanese), the timing of CMV infection (perinatal vs congenital), or factors other than *TLR*s and infant’s functionally immature immune system that are responsible for higher susceptibility to CMV infection. Findings on *TLR4* gene suggested that rs4986790 was not associated with congenital CMV infection in infants (18,20), but that it carries decreased the risk of CMV infection in adults (18). Results on TLR9 rs352140 showed no association with congenital CMV infection in one study on Japanese infants (19), while another study on Caucasian infants showed a significantly higher risk in heterozygotes (20), a finding that should be taken with caution considering the very small number of cases. Another gene, *MBL2*, has been suggested, with studies that showed that heterozygotes (A/O, A being the wild type and O the three SNPs) and homozygotes (O/O) had decreased levels of MBL protein (21,27). In Chinese Han infants XB variant was associated with low *MBL* levels, which represents an increased risk of either acquiring CMV infection or progressing to CMV disease in already seropositive patients (21). In contrast, a study in Caucasian infants showed no significant association in the frequency of MBL2 haplotypes among infected and healthy infants (22).

CMV is a very interesting model for studies on host genetics. First, it is a member of *Herpesvirus* family, which includes a fair share of latent infections, which may not show any symptoms, or symptoms may be triggered by immune system failures (28). In the case of a congenital infection, there is a much lower level of confounding risk, when compared to other infections because of the lack of adaptive immunity and relative unimportance of microbiomes interactions. However, this also suggests that future studies should be based on dyadic analyses, taking into account both the newborn and the mother, in an attempt to better understand disease pathogenesis. We also need to understand the difference between symptomatic infections and latent asymptomatic ones, in order to identify factors that distinguish these two phenomena. Also, it is very likely that different genes are involved in different clinical presentations. While one set of genes might be important for CMV susceptibility, it is very likely that a completely different set of genes might be important for its clinical presentation, which is more linked to the general response of the host to not just CMV, but to other similar pathogens as well. A comparative analysis might help us understand which genes are involved in which pathways, and thus enable better understanding of the disease and its mechanisms. This duality fits well with the proposal that there may be a large number of genetically susceptible and masked hosts in the primary immunodeficiencies (29-31). We must assume a much more complex genetic background for disease development than the majority of previous studies did, and use appropriate tools from the hypothesis-free domain, especially a genome-wide association analysis (32). Even more interesting would be the development of more advanced study designs, which might attribute to better understanding of the issue of infection and re-infection, infection timing, maternal involvement, or even the issue of why some seropositive patients do not develop clinically apparent disease (33-35). All these activities might fit in well with the recent efforts to develop a vaccine and reduce the overall CMV burden (36).

In order to methodologically improve primary studies and provide better evidence for genetic susceptibility to CMV infections, substantial methodological improvements must be made, both on the host side (newborn/child and maternal), as well as the pathogen side. First, controls, selection, and diagnostics must be of the highest standard, as case-control study design is very sensitive to failures of appropriate management of controls. This is often seen in studies that report controls as apparently healthy subjects, without control for the latent and asymptomatic infections. We also need to develop better biomarkers for the disease status assessment (37) and enable the development of true infect-omics studies (38), based on novel high-throughput molecular methods. Better diagnostics is another important measure, coupled with inclusion of maternal information. Lastly, all future studies should adhere to the widely-accepted guidelines such as STREGA (39), and ideally, all the raw data should be made publicly available to facilitate the global use of data and advance progress in CMV pathogenesis understanding.

This systematic review has several limitations. First, we identified only 5 studies as the source of information, which does not allow for wider-scale analyses or for a quantitative synthesis in the form of meta-analysis. In addition, there were only 2 or 3 studies per gene, providing only a very limited insight into the study question. The individual study sample sizes were also very low, substantially reducing analytical power. Another possible limitation lies in the quality of the studies and their methodology and we detected some risk for bias in all the studies. The majority of studies on CMV, after database screening, were focused on post-transplantation status, which is additionally methodologically confounded by primary diagnosis and the pre-transplant medication, therefore reducing comparability of studies. In addition, there were several studies which focused on outcomes of CMV infection such as hearing loss, which was not detected in all participants. This suggests yet another layer of complexity, which is probably partly affected by host genetics and which also reduces the comparability of these studies. For these reasons we narrowed our research question with fewest possible confounders and focused on CMV infection susceptibility in children and congenital cases.

In conclusion, it seems that the exact mechanisms of CMV infection and associated pathogenesis are by far more complex than we expected, and that we need to aim to provide answers for focused level hypotheses, and only then build the knowledge base and understanding of the disease as a whole. Further advancements are possible in better understanding of the molecular level and in immune domain, followed by the validation in human studies. In addition, a case of framework systematic review might serve as the hypothesis-generation source of information and help us understand CMV pathogenesis in much greater detail.
