# Supplementary material for: Host genetics and susceptibility to congenital and childhood cytomegalovirus infection: a systematic review
Source: Croat Med J. 2016 Aug;57(4):321–30. doi: 10.3325/cmj.2016.57.321 (PMC5048223; doi:10.3325/cmj.2016.57.321)
Supplement: Supplementary Table 1 [file CroatMedJ_57_s001.pdf]

Supplementary Table 1. Search terms and details

**PubMed (http://www.ncbi.nlm.nih.gov/pubmed/)**
(SNP[All Fields] OR "polymorphism*"[All Fields] OR "polymorphism, single nucleotide"[MeSH Terms] OR "single nucleotide polymorphism"[All Fields] OR "polymorphism, genetic"[MeSH Terms] OR "genetic polymorphism"[All Fields] OR (("genes"[MeSH Terms] OR "genes"[All Fields] OR "gene"[All Fields]) OR variant[All Fields]) OR "genotype"[MeSH Terms] OR "alleles"[MeSH Terms] OR "alleles"[All Fields] OR "allele"[All Fields]) AND ("Disease Susceptibility"[Mesh Terms] OR "susceptibility"[All Fields] OR "sensitivity"[All Fields] OR "risk"[MeSH Terms] OR "risk"[All Fields] OR severity[All Fields] OR "association"[MeSH Terms] OR "association"[All Fields] OR "mortality"[Subheading] OR "mortality"[All Fields] OR "mortality"[MeSH Terms]) AND ("cytomegalovirus"[MeSH Terms] OR "cytomegalovirus"[All Fields] OR "human herpesvirus 5"[All Fields] OR "cytomegalovirus infections"[MeSH Terms] OR ("cytomegalovirus"[All Fields] AND "infections"[All Fields]) OR "cytomegalovirus infections"[All Fields]) AND ("humans"[MeSH Terms] OR "humans"[All Fields] OR "human"[All Fields]) AND English[lang]
**Web of Knowledge (wok.mimas.ac.uk)**
TOPIC: ((SNP OR gene OR variant OR polymorphism OR genotype OR allele OR genetic) AND (susceptib* OR sensitiv* OR association OR sever* OR mortality OR risk) AND (cytomegalovirus OR cytomegalovirus infection OR human herpesvirus 5) AND human) AND LANGUAGE:(English)
**SCOPUS (http://www.scopus.com/)**
TITLE-ABS-KEY(SNP OR gene OR variant OR polymorphism OR genotype OR allele OR genetic) AND (susceptib* OR sensitiv* OR associat* OR sever* OR mortality OR risk) AND (cytomegalovirus OR cytomegalovirus infection OR human herpesvirus 5) AND (human) AND LANGUAGE(english)
**HuGe Literature Finder (http://www.hugenavigator.net)**
CMV
