# Supplementary material for: Host genetics and susceptibility to congenital and childhood cytomegalovirus infection: a systematic review
Source: Croat Med J. 2016 Aug;57(4):321–30. doi: 10.3325/cmj.2016.57.321 (PMC5048223; doi:10.3325/cmj.2016.57.321)
Supplement: Supplementary Table 2 [file CroatMedJ_57_s002.pdf]

Supplementary Table 2. Domains and grades of CSI score

Domain	Level A grade	Level B grade	Level C grade
**Confounding risk**	No apparent confounding (or possible confounding properly adjusted for) AND no indication of population stratification	Some degree of confounding possible/probable OR study performed in obviously admixed population	Detectable levels of confounding OR indication of strong stratification
**Selection bias risk**	Controls drawn from general population AND satisfy Hardy-Weinberg equilibrium (HWE)	Controls drawn from structured sampling frame (hospital, clinic or health care programme-based) AND in HWE	No description on controls recruitment OR controls fail HWE
**Information bias risk**	I1: Cases status verified by highly specific molecular methods (antigen test, PCR)<br>I2: Controls status verified by highly specific molecular methods (antigen test, PCR)<br>I3: Favourable genotyping quality control estimates given, subset or total dataset replicated	I1: Cases status established on the basis of guidelines, clinical status, or less specific methods (isolation, smears and microbiological cultures)<br>I2: Controls status inferred from medical records only (no history of disease)<br>I3: Partial genotyping quality control results	I1: No clear case definition provided<br>I2: No description of disease status in controls ("healthy" controls)<br>I3: No indication of genotyping reproducibility
